# Medical News and Miscellany

**Published:** 1892-03

**Authors:** 


### Medical News and Miscellany.

**Dr. W. P. Perkins** has removed from Bullard, Texas to El Paso.

**Dr. J. A. Abney** has removed from Lufkin to Lampasas, and Dr. Layton from Lampasas to Beeville.

**Dr. C. L. Orr** has removed from Boz, Ellis county, to Oakville, Live Oak county, Texas.

**J S. Steele, M. D.**, of San Marcos, has just returned from the Vanderbilt with his diploma, and will locate at Llano.

**Dr. Q. C. Smith**, of Austin, was thrown from his buggy on the 8th inst., and considerably disfigured, but is still in the ring.

**Dr. Seth Morris**, Professor of Chemistry in the Medical College, University of Texas, is quite sick at his father's home, in Austin.

**Dr. J. S. Price**, the County Health Officer of Liberty county, was married at Austin, March 10th inst., to Miss Lou Stelfox, of Austin

**Dr. T. B. Bass**, of Terrell, Texas, has been appointed Assistant Physician to the Terrell (State) Lunatic Asylum, vice Dr. F. S. White, made Superintendent at Austin.

**Dr. L. L. Jones**, of Forney, is attending a special course of lectures in New York, and his address is 214, 216 E. 34th Street, where desires the JOURNAL to be sent till June.

**Dr White**, Superintendent State Lunatic Asylum at Austin, wishes to get up a library for the use of patients, and will thankfully receive contributions of new or second hand books and periodicals, especially those with pictures.

**Married.**—At Rusk, Texas, February 24th, Dr. J. W. Thomason, of Huntsville, to Miss Sue Hayes Goree, daughter of Major Thos. J. Goree, of Rusk. The bride and groom went on a trip to New York, and will return to Texas in May.

**Congress** has taken up a bill to compel manufacturers of baking powders who use alum and ammonia in the composition of baking powders to label the cans, so that purchasers may know what they are buying. This is a righteous movement, and the JOURNAL hopes the bill will pass.

---

**Dr. H. L. Fountain**, one of the leading physicians of Bryan, Texas, spent several days in Austin recently *en route* to Llano, the coming Birmingham or Manchester of Texas. Dr. Fountain goes to Llano on a prospecting tour, with a view of investing in property and also of establishing a Sanitarium, as the place is already well known as a health resort. We wish the doctor success.

---

**Sad Accident.**—At Atlanta, Texas, on March 11th, the ten-year-old son of Representative Dr. A. C. Oliver, of Douglasville, this county, while on the way to Atlanta on a wagon load of cotton accidentally fell. The wagon passed over his left leg below the knee, breaking and mashing the limb in a fearful manner. He was brought to Atlanta, and, after receiving medical attention, is resting easy.—*Statesman.*

---

**Mr. Will Robinson**, agent for Colter's Steam Vaporizer, spent some two weeks in Austin, introducing this late invention to the doctors. He is a young man of enterprise and pluck, and having brought proper credentials and endorsements, was well received here. He will visit most of the larger towns, and will call on members of the profession. The JOURNAL bespeaks for him a courteous reception; he is a gentleman, worthy of it, and will appreciate it.

---

**The Journal** regrets to learn that Dr. E. J. Beall, of Fort Worth, met with a serious accident recently while performing a surgical operation. In amputating the leg of a prominent citizen of Callahan county, for chronic destructive tubercular arthritis, he pricked his own finger, thus inoculating himself with septic matter. It resulted in a severe (septic) axillary adenitis which confined the doctor to his bed several weeks. At last account he was out of danger and able to be about.

---

**A Texas Statuette of Hygeia.**—Dr. W. H. Baldinger, of Galveston, has issued a card to the doctors of Texas, requesting them to send him minerals, shells or other products representative of their counties, from which he proposes to construct a "Statuette of Hygeia" to be exhibited at the World's Fair in 1893, and at the Texas Exhibit at Galveston in 1892. There is a very great variety of minerals in Texas, and if the doctors will heed the request Dr. B. should be able to get up a very interesting and variegated "hygeia."

---

**Our Faux Pas**—In the February issue of the JOURNAL we had intended to call attention to a special course at the Chicago POLYCLINIC, beginning on the 28th of March inst., and under the head of "A Rare Opportunity" we gave all the details—including the names of the professors in charge of each branch; but in some unfortunate and unaccountable way we made use of the "Chicago *Post-graduate* School" instead of the "Chicago POLYCLINIC!" We beg here to say that until our attention was called to it we did not even know that there is in Chicago such a thing as a post-graduate school. We know nothing whatever of it, nor of those connected with it. Our remarks were intended for the POLYCLINIC, the professors in which school are all well known, eminent teachers—such as Fenger, Senn, Etheridge, Brown, and others. The article is reproduced this month, *corrected.* Please read. Our apologies are also tendered the POLYCLINIC.

---

**Texas Medical College.**—The April number of the JOURNAL will contain a write-up of the Medical Department of the University of Texas and a resumé of the first year's work. It will be illustrated with engravings of the several professors, and of the College Hospital building. This will be a memorial edition in honor of the annual meeting of the State Association; and it is to be hoped the members will show their appreciation of our enterprise and efforts to please them by a liberal subscription. We expect to be at Tyler in person, partly in the interest of the JOURNAL, and hope to receive a great many new subscribers. Our old friends and subscribers are asked to help us. It will be a memorial edition and a valuable one, a copy of which every physician who feels an interest and pride in the successful inauguration of a high grade medical school in Texas should preserve in his library. Every physician in the State should have a copy. Orders will be received in advance for copies for friends and a discount made on quantities.

---

**A New Summer Medical School.**--The Sewanee Medical School, the Medical Department of the University of the South, will begin its first course on Monday, April 11, 1892, and close Aug. 27,—a session of twenty weeks. The faculty consists of seven professors, all eminent teachers, and a complete course in every branch of medicine will be given. This course will cover the period when other medical schools are closed, and the climate of Sewanee, on the celebrated Cumberland mountain plateau, being remarkable for its salubriety, cool and inviting, will afford much comfort and freedom from heat and dust to those who wish to prosecute their medical studies during the summer months. Sewanee is equally removed from the malaria of the South and danger of pulmonary diseases of the North, and to spend the summer amid the cool shades of the fine forests of the college grounds must be very delightful. Dr. H. W. Blanc, late of New Orleans, so well and favorably known to the profession and people of the South, is Dean of the college, and Professor J. S. Cain of Nashville, equally popular with the Southern boys, is Professor of Medicine. Physicians of over five years practice on payment of the matriculation fee will be admitted to all the lectures free, while those of less than five years since graduation will be charged half price.

The University of the South, it will be remembered, is an Episcopal school; but that has nothing to do, of course, with the teaching of medicine. For catalogue address Dr. H. W. Blanc, Sewanee, Tenn., mentioning the JOURNAL.

If there was an opening for any other medical school in the country it was for a high grade summer school, and the management of this enterprise have been most fortunate in the selection of a location and accessories, as well as in the selection of a Faculty.

---

**A Rare Opportunity.**—The *Chicago Polyclinic* will begin, on the 28th day of March, inst., *a special course* of two weeks in medicine and surgery. Drs. Nicholas Senn, J. B. Hamilton, Belfield and Fenger, all eminent and distinguished as teachers, will conduct the surgical clinics, while Drs. Ethridge, the Dean of the Rush Medical College, with Bauga and Hernotin will have charge of the gynecological work. Drs. Fletcher Ingalls and M. B. Brown will conduct the throat clinic. Other distinguished clinicians will be connected with the course, and in every department the work will be carried along, the very latest phases of practice being exemplified and illustrated. Amongst them we may mention Futterer, whose work on bacteriology recently electrified Germany; Hotz and Colburn, whose eye work is unsurpassed for neatness of technique, and successful results. Those who go to this course must expect to work, if they would profit by the opportunity; it will be an all-day matter each day of the two weeks, the clinics beginning at 8:30 a. m., and closing at 10 p. m.

This will be the fourth of these special working courses. They have been well patronized, and are especially intended for earnest men, who desire to brush up, and whose time is limited; excellent for the general practitioner who cannot spare six or eight weeks from home. The eyes of the world are turned to Chicago now, and railroad travel is cheap. Dr. M. B. Brown will be remembered as an old Texas confrere, formerly of Galveston. He will make the visit pleasant and profitable for the Texans who avail themselves of this opportunity. Write to him.

### Antikamnia—(Opposed to Pain).

Our attention has been frequently called during the past year, to the claims made by the progenitors of Antikamnia, and as a result, after careful investigation, we submit the following as a compendium of our examination of its pathological and physiological action :

The therapeutic properties are antipyretic, antithermic, analgesic and anodyne. Klemerer, of Germany, makes a distinction between antipyretics and antithermics. He says, "Antithermics act only on the temperature; that is, they influence its reduction, while antipyretics influence the *cause* of the high temperature.

Fever is an acute derangement of all function, the most important of which are acceleration of the heart's beat, and disturbance of the circulation; nervous disturbance; elevation of the bodily temperature; disturbance of nutrition, including secretion.

These four groups of symptoms may have one or two relations. One condition may be the cause of the other, or they may all be simply the result of a common cause. The nervous disturbances of fever may be summed up as a paresis or convulsions, stupor, coma, or delirium.

JERGENSON has found that there is a regular diurnal variation of temperature in health, precisely similar to that which is known to occur in fever, thus the 24 hours is, as far as human temperature is concerned, divided into a diurnal and nocturnal period.

BURDON SANDERSON says: "The only material difference between the conditions is that in fever the normal is 3.267° F. higher."

In health, there is in man a fixed mean and a normal temperature, having a regular rhythm, and this variation is beyond the control of all disturbing causes, which do not force the organism beyond the condition of health. The maintenance of the normal temperature and its rhythm is dependent upon the nervous system, which within certain limits controls both the production and dissipation of animal heat.

So far as our present knowledge goes, the chief factor in controlling heat dissipation, is the vaso-motor nerves, including in man such nerves as control sweat secretions; these nerves being able, by contracting the capillaries of the surface of the body, and by drying the secretions of the skin, to reduce the loss of heat to a minimum, and by a reverse action to increase it to a maximnm. The only nerve centre proven to exist, capable of influencing the heat production without affecting the general circulation, is situated in the pons varolii, or above it, and whilst it may be a muscular vaso-motor centre, it is probably an "inhibitory heat centre." Of whichever nature it may be, it must act through subordinate centres situated in the spinal cord.

In fever, vaso-motor paralysis, when produced, is followed by an immediate fall of temperature. Fever is, therefore, a state in which the depressing poison, or a depressing peripheral irritation, acts upon the nervous system, which regulates the production and dissipation of animal heat. Owing to its depressed state, the inhibition centre does not exert its normal influence upon the system, and consequently tissue change goes on at a rate which results in the production of more heat than normal, and an abnormal destruction and elimination of the materials of the tissue. At the same time the vaso-motor and other heat dissipating centres are so benumbed that they are not called into action by their normal stimulus—elevation of the general bodily temperature, and do not provide for throwing off the animal heat until it becomes so excessive as to call into action, by its excessive stimulation, even their depressed forces. The nerve centres, in some cases, seem to be completely inhibited. Antikamnia removes the pressure, by dilating the capillaries and the other vascular vessels, thus causing local congestion to disappear. It reduces the pulse rate, thereby slowing the heart. It controls the vaso-motor nerves, besides calming the whole nervous system, and thus has a general soothing effect. It is a valuable remedy as an antithermic; its action in this regard is well marked, sometimes reducing the temperature 2° to 3° F. in a few hours. It seems to have a better effect on the high evening temperature than upon the high diurnal temperature. An extreme degree of fever, with or without complicatlons, is dangerous, and must be controlled; in addition to the direct substraction of heat by cold applications, we must, with caution, have recourse to antipyretic remedies. A distinction must be drawn between fever and its pathogenic agent. Such an antipyretic as Antikamnia may not act on this agent, but may have an independent action, therefore, have oniy a transitory effect, or it may influence this agent in the some manner that quinine does the germ of malaria or influenza.

An additional advantage gained in typhoid fevers and all gastroenteric fevers by the administration of Antikamnia in moderate doses, is that the alimentary canal is rendered alkaline, and kept in an antiseptic condition, and this is a most important condition to maintain in the treatment of all fevers.

The best results are obtained with Antikamnia when exhibited in small doses, repeated at proper intervals, and the most desirable vehicle is sherry wine or diluted brandy.

The duration of the effect of Antikamnia is longer than that produced by any of the other coal tar derivatives. It also seems indisposed to produce sub-normal temperature, as some of the others do.

In the pyrexia produced by exposure to the rays of the sun, which is common in India and in our large cities during the summer solstice, Antikamnia, in addition to cold douches, is the best remedy. Antikamnia reduces temperature by increasing radiation of heat from the body, and diminishing heat production. It stimulates the glandular system, particularly the sudorific glands. In many cases its action as a diaphoretic is phenomenal.

In some cases it has marked action on the mammary glands, producing an increase in the flow of milk. Antikamnia can be given to children without any ill effects, and is a reliable remedy.

In pertussis, it keeps the paroxysms in check, and makes the patient more comfortable than any remedy we have. The cyanosis induced by its administration is *nil*, unless there is a peculiar idiosyncrasy, which is found sometimes, producing manifest heart disturbance. These are to be overcome by stimuli, or intravenous injections of salt. Antikamnia acts admirably in the after-pains of labor, in dysmenorrhœa, hemicrania, migraine, ordinary sick or nervous headache, in the pains of locomotor ataxia, the various neuralgias, epilepsy, and in the aching pains produced by la grippe and dengue. It exerts a decided beneficial influence in bronchial and pneumonic troubles, as well as the fever of phthisis.

It acts as an analgesic by obtunding the sensibilities of the vaso-motor and sensory nerves. It seems to tranquilize the ganglionic centers of the whole nervous system, and has but slight action on the brain. We mean by this, that it does not stupify or produce unconsciousness. It seems to have no disturbing influence on the kidneys. It has a happy effect in nearly all neurotic troubles, and is destined to occupy a permanent position in therapeutics.

Antikamnia is of the Amido-Benzole series, in combination, and is much to be preferred to any other of this class of derivatives.—*Exchange.*

